# Reassortment Between Divergent Strains of Camp Ripley Virus (*Hantaviridae*) in the Northern Short-Tailed Shrew (*Blarina brevicauda*)

**DOI:** 10.3389/fcimb.2020.00460

**Published:** 2020-09-09

**Authors:** Schuyler W. Liphardt, Hae Ji Kang, Satoru Arai, Se Hun Gu, Joseph A. Cook, Richard Yanagihara

**Affiliations:** ^1^Museum of Southwestern Biology and Department of Biology, University of New Mexico, Albuquerque, NM, United States; ^2^Department of Pediatrics, John A. Burns School of Medicine, University of Hawaii at Manoa, Honolulu, HI, United States; ^3^Infectious Disease Surveillance Center, National Institute of Infectious Diseases, Tokyo, Japan

**Keywords:** *Hantaviridae*, host switching, reassortment, viral evolution, viral phylogenetics

## Abstract

Genomic reassortment of segmented RNA virus strains is an important evolutionary mechanism that can generate novel viruses with profound effects on human and animal health, such as the H1N1 influenza pandemic in 2009 arising from reassortment of two swine influenza viruses. Reassortment is not restricted to influenza virus and has been shown to occur in members of the order *Bunyavirales*. The majority of reassortment events occurs between closely related lineages purportedly due to molecular constraints during viral packaging. In the original report of Camp Ripley virus (RPLV), a newfound hantavirus in the northern short-tailed shrew (*Blarina brevicauda*), phylogenetic incongruence between different genomic segments suggested reassortment. We have expanded sampling to include RPLV sequences amplified from archival tissues of 36 northern short-tailed shrews collected in 12 states (Arkansas, Iowa, Kansas, Maryland, Massachusetts, Michigan, Minnesota, New Hampshire, Ohio, Pennsylvania, Virginia, Wisconsin), and one southern short-tailed shrew (*Blarina carolinensis*) from Florida, within the United States. Using Bayesian phylogenetic analysis and Graph-incompatibility-based Reassortment Finder, we identified multiple instances of reassortment that spanned the *Hantaviridae* phylogenetic tree, including three highly divergent, co-circulating lineages of the M segment that have reassorted with a conserved L segment in multiple populations of *B. brevicauda*. In addition to identifying the first known mobatvirus-like M-segment sequences from a soricid host and only the second from a eulipotyphlan mammal, our results suggest that reassortment may be common between divergent virus strains and provide strong justification for expanded spatial, temporal, and taxonomic analyses of segmented viruses.

## Introduction

Genomic reassortment is an important evolutionary mechanism by which phylogenetically distinct, segmented viral strains that co-infect a host cell may shuffle gene segments to generate new viral genotypes (Vijaykrishna et al., 2015; Lowen, 2018). Reassortment is possible for any segmented virus and occurs during the packaging stage of viral replication where individual segments from two or more strains are combined into a single virion. While a majority of reassortment events are thought to result in deleterious combinations and subsequent reduction in fitness, reassortment on occasion can lead to novel combinations that can result in increased fitness (McDonald et al., 2016). However, the extent of reassortment and associated clinical implications, such as increased pathogenicity, are not well-defined. In influenza A virus, reassortant strains have been repeatedly documented to increase virulence compared to the respective parental strains (White and Lowen, 2018). For example, the H1N1 strain responsible for the 2009 influenza A epidemic was a reassortant composed of several divergent strains of influenza virus, including at least two from swine, and one each from birds and humans (Trifonov et al., 2009). Reassortment is not restricted to influenza virus. It has been suggested that a large number, possibly most, currently recognized members of the order *Bunyavirales* are reassortants (Briese et al., 2013). This includes evidence of reassortment in disease-causing viruses, such as Crimean-Congo hemorrhagic fever virus (Hewson et al., 2004), Rift Valley fever virus (Liu et al., 2017), Ngari virus (Lowen, 2018), and many hantaviruses (Klempa, 2018).

Possible implications to human health posed by viral reassortment may be profound, given that global movement of people and changing species distributions due to environmental disruption may increase novel interactions of divergent viruses (Carlson et al., 2017, 2020). Understanding the extent of viral reassortment among wild reservoir host populations, as opposed to *in vitro* experiments, and the effectiveness of segment molecular incompatibilities in constraining successful reassortment, is dependent on spatial and temporal screening of diverse wild hosts at the community and population levels, as well as concerted efforts to produce genomic level sequences for comparative analyses.

Viruses of the family *Hantaviridae* contain three genomic segments named for their relative size, small (S), medium (M), and large (L), which encode the nucleocapsid protein, glycoprotein precursor, and RNA-dependent RNA polymerase, respectively (Plyusnin et al., 1996; Abudurexiti et al., 2019). Klempa (2018) provides a summary of the role reassortment has played in the evolution of hantaviruses across multiple temporal scales. For example, Bruges virus in the European mole (*Talpa europaea*) is likely the result of one or more ancient reassortment events (Laenen et al., 2018). In contrast, other studies suggest recent reassortment events in Sin Nombre virus harbored by the deer mouse (*Peromyscus maniculatus*) (Henderson et al., 1995; Li et al., 1995; Black et al., 2009) and Puumala virus in the bank vole (*Myodes glareolus*) (Razzauti et al., 2008, 2009). Briese et al. (2013) suggested that reassortment may be quite common and that all members of the order *Bunyavirales*, of which *Hantaviridae* is a member, could be reassortants or descendants of historical reassortment events.

Camp Ripley virus (RPLV) is a hantavirus originally discovered in the northern short-tailed shrew (*Blarina brevicauda*) (Order Eulipotyphla, Family Soricidae) (Arai et al., 2007). Bennett et al. (2014) suggested that the S segment of RPLV could be the result of recombination due to its ambiguous placement in the hantavirus phylogenetic tree; however, that assessment was based on sequence coverage from a single sample that limited insight into the evolutionary history of RPLV. With expanded sampling of RPLV from multiple populations and new statistical approaches, we have been able to develop a clearer picture of host switching and reassortment within RPLV. However, much more extensive sampling across populations and geography of *B. brevicauda* is still necessary to achieve a truly complete history.

## Materials and Methods

### Ethics Statement

All field procedures for trapping of shrews and well-established protocols for processing and preserving their tissues were reviewed and approved by the Institutional Animal Care and Use Committee of the University of New Mexico, under protocol number 19-200908-MC.

### Sampling and Sequencing

**Figure 1:**
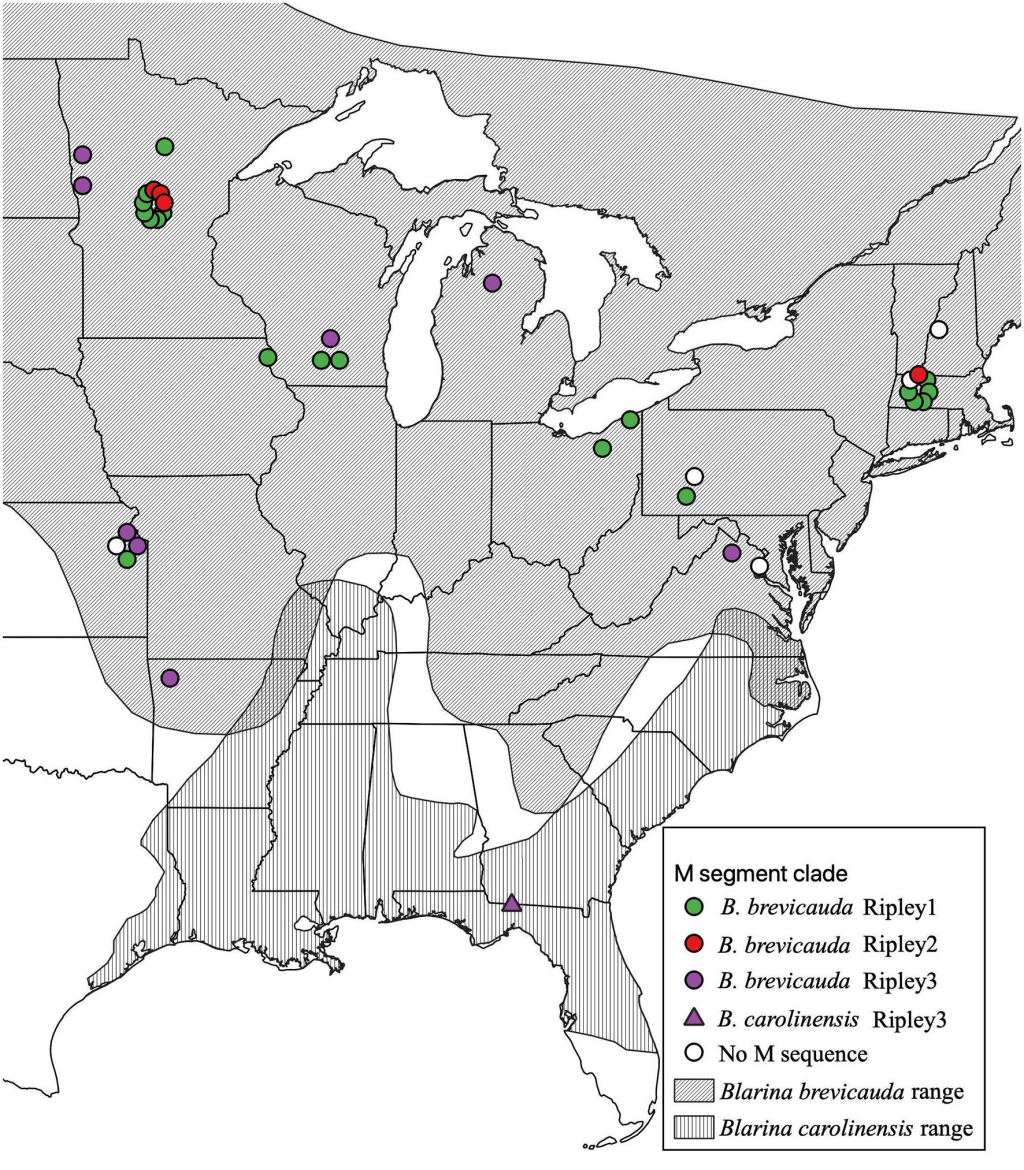
Geographic distribution of RPLV M-segment clades according to collection sites of *Blarina brevicauda* and *Blarina carolinensis* in the United States. RPLV M-segment clades are represented by colored circles (green for Ripley1, red for Ripley2, and purple for Ripley3). White circles represent RPLV samples for which L- and/or S-segment sequences are available, but M segment sequences could not be amplified. Species distributions were acquired from the international union for conservation of nature website. Species distributions were acquired from the International Union for Conservation of Nature website (IUCN, 2008; NatureServe, 2008).

Total RNA was extracted from liver and/or lung tissue, using the PureLink Micro-to-Midi total RNA purification kit, from 101 *B. brevicauda* and 10 southern short-tailed shrews (*B. carolinensis*) archived at the Museum of Southwestern Biology at the University of New Mexico (Table 1). Shrews were collected between 1980 and 2001 and represent sampling across a large portion of the distributions of both species in the United States (Figure 1). Complementary DNA (cDNA) was synthesized using the SuperScript III First-Strand Synthesis System (Invitrogen, San Diego, CA) with a universal oligonucleotide primer (5′-TAGTAGTAGACTCC−3′) designed from the conserved 3′-end of the S, M, and L segments of hantaviruses (Song et al., 2007).

**Table 1:** Museum of Southwestern Biology (MSB) catalog numbers, GenBank accession numbers, and tissue type for Camp Ripley virus.

**State**	**County**	**Year**	**MSB number**	**S**	**M**	**L**	**Tissue**
Arkansas	Washington	14-Mar-1982	MSB49712	MT377511 (885)	MT377558 (1480)	MT377528 (347)	Liver
Florida	Leon	18-Jun-1983	MSB53277		MT377562 (205)		Liver
Iowa	Allamakee	24-Aug-1994	MSB73580		MT377535 (1154)	MT377512 (347)	Lung
Kansas	Douglas	11-Oct-2001	MSB151832		MT377555 (1187)		Lung
		11-Oct-2001	MSB151836		MT377542 (875)	MT377523 (347)	Lung
		11-Oct-2001	MSB151838		MT377556 (1187)	MT377522 (347)	Lung
		11-Oct-2001	MSB151840			MT377521 (347)	Lung
Maryland	Charles	22-Sep-1997	MSB92373			MT377518 (347)	Liver
Massachusetts	Franklin	10-Jul-1982	MSB47876		MT377549 (1104)	MT377531 (347)	Liver
		12-Jul-1982	MSB47879		MT377551 (1082)	MT377533 (347)	Liver
		13-Jul-1982	MSB47882	MT307509 (394)			Liver
		11-Jul-1982	MSB47883		MT377550 (1099)	MT377532 (347)	Liver
		12-Jul-1982	MSB47887	MT307508 (400)	MT377552 (1101)		Liver
		12-Jul-1982	MSB47890		MT377561 (1105)		Liver
		13-Jul-1982	MSB47891	KF958463 (1028)	MT377553 (1103)		Liver
Michigan	Crawford	9-Jul-1999	MSB92437		KF958467 (2345)		Liver
Minnesota	Clay	31-Jul-1983	MSB53249		MT377554 (886)		Liver
		31-Jul-1983	MSB53251		MT377560 (1130)	MT377519 (347)	Liver
	Cass	28-Jul-1983	MSB53254	MT377507 (783)	MT377548 (1370)	MT377530 (347)	Liver
	Morrison	20-Aug-1998	MSB89858		MT377536 (1155)	MT377513 (347)	Lung
		21-Aug-1998	MSB89859		MT377537 (1390)	MT377514 (347)	Lung
		20-Aug-1998	MSB89861		MT377538 (1390)	MT377515 (347)	Lung
		20-Aug-1998	MSB89862		MT377539 (1153)	MT377534 (347)	Lung
		20-Aug-1998	MSB89863	FJ790772 (330)	EF540774 (2755)	EF540771 (6551)	Lung
		20-Aug-1998	MSB89864		MT377540 (1155)	MT377516 (347)	Lung
		21-Aug-1998	MSB89866	MT377505 (276)	EF540775 (1717)	EF540772 (300)	Lung
		19-Aug-1998	MSB89867		MT377541 (1041)	MT377517 (347)	Lung
		21-Aug-1998	MSB90845	KF958464 (900)	EF540773 (2077)	KF958465 (6551)	Lung
New Hampshire	Grafton	1-Sep-1998	MSB151869			MT377525 (347)	Lung
Ohio	Summit	21-Aug-2001	MSB151834		MT377543 (1390)	MT377524 (347)	Lung
	Ashtabula	30-Jun-1980	MSB43407		MT377546 (1102)		Liver
Pennsylvania	Indiana	2-Oct-2001	MSB151849	MT377506 (425)		MT377527 (347)	Lung
	Westmoreland	5-Jul-1983	MSB53264	MT307510 (1100)	MT377547 (884)	MT377529 (347)	Liver
Virginia	Fauquer	2-Apr-1994	MSB76255		MT377559 (1479)		Liver
Wisconsin	Dane	21-Sep-2000	MSB151873		MT377557 (1187)		Lung
		21-Sep-2000	MSB151874		MT377545 (1390)	MT377520 (347)	Lung
		21-Sep-2000	MSB151878		MT377544 (1390)	MT377526 (347)	Lung

*Numbers in the parentheses represent the length of sequence recovered for that segment*.

Gene amplification was carried out in 20-μL reaction mixtures containing 250 μM dNTP, 2 mM MgCl_2_, 1 U of AmpliTaq polymerase (Roche, Basel, Switzerland), and 0.25 μM of oligonucleotide primers, designed from highly conserved regions of previously identified soricid-borne hantaviruses. A listing of the oligonucleotide primers used to amplify the S, M, and L segments is provided in Supplementary Table 1. Initial denaturation was followed by touchdown PCR cycling (two-degree step-down annealing from 48 to 38°C for 40 s) and elongation at 72°C for 1 min, then 32 cycles of denaturation at 94°C for 40 s, annealing at 42°C for 40 s, and elongation at 72°C for 1 min, in a GeneAmp PCR 9700 thermal cycler (Perkin-Elmer, Waltham, MA, USA). Amplified products were separated by electrophoresis on 1.5% agarose gels and purified using the QIAQuick Gel Extraction Kit (Qiagen, Hilden, Germany). DNA was sequenced directly using an ABI Prism 377XL Genetic Analyzer (Applied Biosystems Inc., Foster City, CA, USA).

### Phylogenetic Analysis

Additional sequences representative of all currently recognized species of *Hantaviridae* were downloaded from GenBank (Supplementary Table 2). Nucleotide alignments were generated for each segment using MAFFT v7.402 (Multiple Alignment using Fast Fourier Transform) (Katoh and Standley, 2013) with the Smith-Waterman algorithm (–localpair and –maxiterate 1000). Alignments were visually inspected in Geneious v. 8 (https://www.geneious.com). The 3′- and 5′-ends that were poorly aligned and had limited coverage across samples were trimmed to ensure analysis of homologous regions. This process was repeated for the amino acid translation of each segment to address possible artifacts introduced by high levels of nucleotide variation and potential homoplasy between deeply divergent sequences. Bayesian phylogenetic inference was performed for nucleotide and amino acid alignments in MrBayes 3.2.6 (Ronquist et al., 2012) with a mixed model of evolution and a gamma distribution with the command “lset nst = mixed rates = gamma” and run for 10,000,000 generations with trees sampled every 1,000 generations. Convergence was verified by inspecting that standard deviation of split frequencies between runs was under 0.01, as recommended, with trees sampled every 1,000 generations. Convergence was tested using Tracer v.1.7.1 (Rambaut et al., 2018) to verify the effective sample size for the posterior was above 200. A burn-in of 25%, as recommended (Ronquist et al., 2012), was used prior to generating a 50% majority rule consensus tree. Means of raw p-distances between groups and within groups were calculated on the amino acid alignment in MEGA7 (Kumar et al., 2016).

### Tests of Reassortment

Reassortment was tested for the M and L segments using the Graph-incompatibility-based Reassortment Finder program (GiRaF version 1.01) (Nagarajan and Kingsford, 2011) with the tree files produced by MrBayes as input. The tree files for each segment were used for graph-mining to determine gene segments with incompatible phylogenies. A reduced dataset was used for analysis in GiRaF to accommodate the requirement of identical taxa between trees. A 25% burn-in, 50% cull, all candidate set, non-star bicliques, and single bicliques options were used for the GiRaF analysis. While GiRaF was originally written for reassortment analysis of influenza viruses, it is broadly applicable to any segmented virus capable of reassortment. A tanglegram of proposed reassorted sequences was visualized in R with the package “phytools” (Revell, 2012). As a secondary test of reassortment the program RDP4 was used (Martin et al., 2015). Samples with sequences for both the L and M segment were concatenated with the S segment, when available. Reassortment breakpoints were tested by using the RDP, GENECONV, Bootscan, Maxchi, Chimera, SiSscan, and 3Seq methods. The highest possible *p*-value for accepting a reassortment event was set to 0.05 and all other parameters set to default.

## Results

### Phylogenetic Analysis and Tests of Reassortment

**Figure 2:**
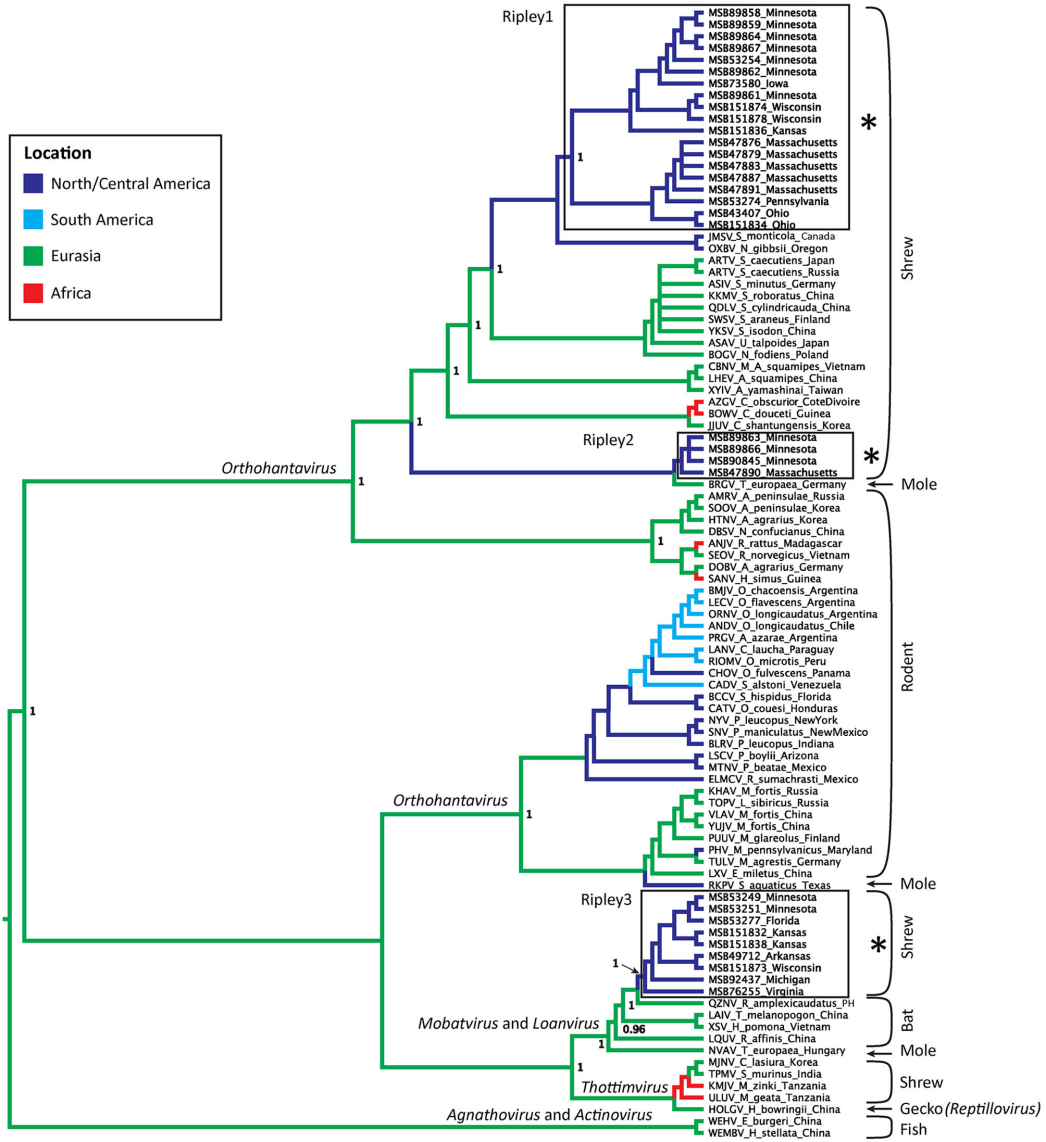
Bayesian phylogeny inferred for the M segment. Branch colors represent geographic origin of the reservoir host and associated virus. Host taxa are indicated (right), with proposed hantavirus genera labeled on respective branches. RPLV clades are boxed, labeled, and indicated with an asterisk. Posterior support values over 0.95 are labeled.

Virus sequences were recovered for 36 *B. brevicauda* and one *B. carolinensis* (Figure 1 and Supplementary Table 3). Phylogenies generated independently for all three segments showed differing levels of incongruence. Trees inferred from the M segment recovered three distinct, deeply paraphyletic clades across the *Hantaviridae* (Figures 1, 2). The largest clade recovered was predictably part of the *Orthohantavirus* clade that includes other eulipotyphlan-borne hantaviruses (harbored by shrews and moles) from both the Old World and New World and represents the majority of our samples (labeled Ripley1). The Ripley1 clade was further subdivided into two well-supported subclades that roughly encompassed the geographic distribution of *B. brevicauda*, with an eastern subclade of shrews from Ohio, Massachusetts, and Pennsylvania, and a western subclade from Minnesota, Iowa, Wisconsin, and Kansas. This East-West division was also reflected in previously generated host phylogenies inferred from cytochrome *b* (Brant and Orti, 2003). The second RPLV clade (Ripley2) was composed of M-segment sequences from *B. brevicauda* collected in Minnesota and Massachusetts, but this segment unexpectedly formed a monophyletic clade with Bruges virus from *Talpa europaea* from Germany. The third RPLV clade (Ripley3) was quite distinct from other RPLV sequences and even more unexpectedly was placed in the genus *Mobatvirus*, sister to Quezon virus from the Geoffroy's rousette (*Rousettus amplexicaudatus*), a pteropodid bat from the Philippines. The Ripley3 clade consisted of M-segment sequences from *B. brevicauda* collected in Arkansas, Wisconsin, Minnesota, Kansas, Michigan, and Virginia, and included the only sequences recovered from *B. carolinensis* collected in Florida. These sequences represented the first known mobatvirus-like M segments recovered from a North American soricid host and only the second from a eulipotyphlan, with the other being Nova virus that co-circulates with Bruges virus in the European mole (Kang et al., 2009; Laenen et al., 2018). Mean within-group amino acid p-distances for each clade in the M segment ranged from 2% to 4% divergence. Mean between-group distances were much larger, however, with 70% amino acid sequence identity between Ripley1 and Ripley2. Ripley3 shared 47% amino acid sequence similarity with Ripley1 and 49% with Ripley2.

**Figure 3:**
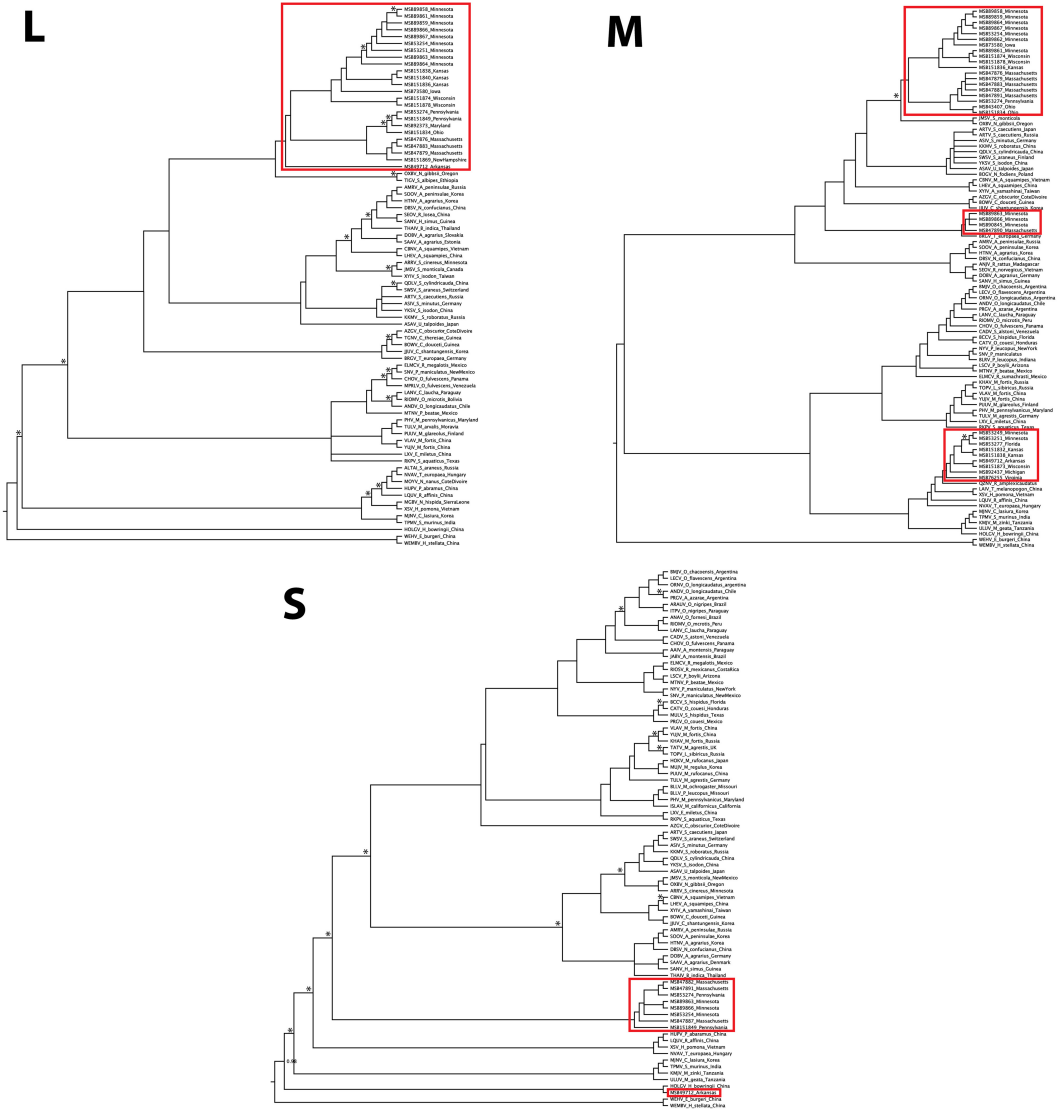
Discordance of Camp Ripley virus across genomic segments. Phylogenies were inferred using Bayesian probability in MrBayes. Nodes with posterior probabilities <0.8 are marked with an asterisk. Camp Ripley representative samples are boxed in red.

The L-segment phylogenies did not match the pattern of diversification seen in the M segment, with all L-segment samples forming a single, well-supported monophyletic clade within *Orthohantavirus* (Figure 3). That clade was composed of virus segments representative of each clade seen in the M segment, however, the L-segment phylogeny placed MSB49712, the single M segment Ripley3 representative, as basal to the rest of RPLV. RPLV formed a sister relationship with Oxbow virus from the American shrew mole (*Neurotrichus gibbsii*) and Tigray virus from the Ethiopian white-footed mouse (*Stenocephalemys albipes*), and in turn were part of the well-supported clade of the genus *Orthohantavirus*. Within the larger clade of RPLV variation was rather low with a mean p-distance about 1.5% divergence. However, when compared to MSB49712, the mean distance was 11%.

Although our sampling for the S segment was less complete than for the M and L segments, we recovered two distinct RPLV clades (Figure 3), with the major S-segment clade containing representatives of both Ripley1 and Ripley2 from the M-segment phylogeny. The one representative from Ripley3 in the S-segment phylogeny, MSB49712 from Arkansas, was related to the Hainan oriental leaf-toed gecko virus (HOLGV), a non-mammalian borne hantavirid-like virus from China, sharing only 43% amino acid sequence similarity with the rest of RPLV. Interestingly, the placement of HOLGV in both the M- and L-segment phylogenies disagreed with the findings in the original report of HOLGV (Shi et al., 2018).

GiRaF analyses identified 11 candidate sets of reassortment with high confidence (>0.95). Of these, nine contained only sequences from RPLV (Supplementary Table 4). Additionally, all RPLV included in the reduced dataset for GiRaF were consistently identified as reassortants. However, our GiRaF settings were set to the highest sensitivity to detect reassortment across the phylogeny, which also resulted in detection of reassortment among subclades within Ripley1 due to incongruence of internal branching structure toward the tips (Supplementary Figure 1). RDP4 identified six samples as likely reassortants with significant *p*-values depending on the method used (Supplementary Table 5). The six identified samples represented all three RPLV clades in the M-segment phylogeny. The breakpoints identified as the sites of reassortment corresponded to the concatenation points between the L, M, and S segments (Supplementary Table 6).

## Discussion

Reassortment among divergent viral strains can catalyze rapid evolution and result in serious consequences for human health. Benign parental strains, when reassorted, can introduce novel interactions with host systems and increase the ability to evade the host immune system or the likelihood of a virion to enter a host cell, leading to increased virulence (Vijaykrishna et al., 2015; McDonald et al., 2016). However, reassortment has been generally thought to be restricted to closely related viral strains due to molecular incompatibilities that inhibit reassortment among highly divergent viruses *in vivo* (McDonald et al., 2016; Klempa, 2018; White and Lowen, 2018). In this study, we show a possible example of reassortment between deeply diverged viruses differing by 30–50% in the amino acid sequence of the M segment. While our understanding of how common reassortment between distantly related hantaviruses is limited, this study suggests that such events might not be so restrained. However, our ability to fully explore this for RPLV is limited by the lack of sequence representation for all segments, as well as by only partial coverage across the RPLV genome.

The hantavirus species demarcation criteria, published in the Ninth Report of the International Committee on Taxonomy of Viruses (King et al., 2012), which required at least a 7% amino acid sequence difference in the complete nucleocapsid and glycoprotein complex, have been abandoned. And DivErsity pArtitioning by hieRarchical Clustering (DEmARC) analysis of the coding regions of the complete S- and M-segment sequences has been used to objectively establish taxonomic classification of the family *Hantaviridae* since 2017 (Laenen et al., 2019). The limitations of our data set (that is, lacking the full-length S- and M-segment sequences of hantavirids from *B. brevicauda* and *B. carolinensis*) do not allow definitive conclusions about whether or not the three RPLV clades represent three different hantavirus species.

Co-circulation of distinct hantaviruses has been reported previously in other eulipotyphlan species, including Seewis and Altai-like viruses in the Eurasian common shrew (*Sorex araneus*) in Finland (Ling et al., 2014), Bruges and Nova viruses in the European mole in western Europe (Laenen et al., 2016), and most recently, Seewis, Altai, and Altai-like viruses co-circulating in sympatric populations of Eurasian common shrew, Laxmann's shrew (*S. caecutiens*), and flat-skulled shrew (*S. roboratus*) in Hungary and Russia (Kang et al., 2019). Here, we report three distinct M-segment lineages of RPLV co-circulating in *B. brevicauda* in the United States. However, co-circulation of distinct viruses within a single species does not necessarily indicate either current or historical reassortment. For example, despite Bruges and Nova virus co-infections of the same host individual on multiple occasions (Laenen et al., 2018), evidence of reassortment between the two viruses has yet to be documented. That is not the case for Seewis and Altai-like viruses co-circulating in shrews where phylogenetic disagreement between segments suggests an ancient history of host-switching and reassortment although this has yet to be tested statistically (Ling et al., 2014; Kang et al., 2019).

We recovered a similar pattern of disagreement between phylogenies for RPLV. Incongruencies in tree topology were found across all three segments of the RPLV genome. Further, the L-segment monophyly within RPLV was consistent with a model of classical co-diversification with its shrew host, whereas the M segment was composed of at least three unique histories suggesting multiple instances of successful host switches followed by subsequent reassortment of the L and S segments. Our sampling across genes limits our ability to more robustly test the history of reassortment in this system. Two samples that do have sequences for the L, M, and S segments, MSB89866 and MSB89863, were identified by RDP4 as containing the M segment from the Ripley2 clade and the L and S segments from representatives of the Ripley1 clade. This would follow the pattern of reassortment within *Hantaviridae* of the L and S segments packaged together with a divergent M segment, although this is not always the case (Klempa, 2018). Expanded sampling across the entire RPLV genome and more individual hosts will be necessary to adequately address this question.

Our analysis of the L segment was limited to a relatively short section (347 bp), potentially affecting the reliability of phylogenetic inference. Still, while deeper nodes lack adequate posterior support, RPLV was well supported as distinct and monophyletic in the L-segment phylogeny (Figure 3). This pattern is difficult to explain biogeographically considering the depth of divergence and geographic distance between the mammalian hosts of the viruses that are sister to each RPLV clade; for example, Bruges virus and Ripley2 (Figure 3). It is possible that the Ripley2 and Ripley3 clades represent ancient lineages that predate the migration of the ancestor of *Blarina* to North America and have since been maintained. We find this scenario unlikely given the deep history of host colonization of North America (Brant and Orti, 2003), coupled with the high mutation rate of hantaviruses that should have a monophyletic lineage of hantaviruses in *Blarina*, which is not what we see. Another possibility is that the placement of the Ripley2 and Ripley3 clades in the M-segment phylogeny is strictly stochastic or an artifact of long-branch attraction among deeply divergent viruses. However, this scenario does not address how such deeply divergent strains are maintained within a single species. Lastly, the Ripley2 and Ripley3 clades could represent a relatively recent host switch to *B. brevicauda*. Implicit in this explanation is that because geography precludes direct contact and transmission between host species there is likely a significant amount of hantavirus diversity that has yet to be discovered, which would help us better interpret these biogeographical gaps. It is likely that *B. brevicauda* is the primary host of RPLV (Ripley1 clade) and that the Ripley2 and Ripley3 clades are the result of recent host-switching events and subsequent reassortment from unknown hantaviruses yet to be discovered. It is important to note, however, that the topologies recovered here, specifically for the L and S segments, could be prone to bias and artifacts during phylogenetic inference due to the relatively short L-segment sequences and the limited S-segment representation.

Overall, our data suggest that reassortment among highly divergent viral strains may be rather common. Evidence of reassortment between deeply divergent viruses highlights the urgency of more extensive sampling of the global virome (French and Holmes, 2020) and hantaviruses, in particular, across diverse mammalian and non-mammalian taxa, including recently discovered hantavirid-like viruses recovered from reptiles, fish, and mosquitoes (Li et al., 2015; Shi et al., 2018). The diversity and evolutionary dynamics of hantaviruses remain limited, as does knowledge of the complete suite of organisms that host them. Continued research focused on increasing breadth of taxa examined, as well as population level studies within individual host species, is required if we are to develop a more complete history of the origin and diversification of the *Hantaviridae* family.

## Data Availability Statement

The datasets presented in this study can be found in online repositories. The names of the repository/repositories and accession number(s) can be found in the article/Supplementary Material.

## Ethics Statement

The animal study was reviewed and approved by the Institutional Animal Care and Use Committee of the University of New Mexico, under protocol number 19-200908-MC.

## Author Contributions

SL, JC, and RY wrote and edited the manuscript. SL performed phylogenetic and statistical analyses. HK, SA, and SG generated the sequence data and assisted in editing the manuscript. All authors contributed to the article and approved the submitted version.

## Conflict of Interest

The authors declare that the research was conducted in the absence of any commercial or financial relationships that could be construed as a potential conflict of interest.
