# Supplementary material for: Reassortment Between Divergent Strains of Camp Ripley Virus (Hantaviridae) in the Northern Short-Tailed Shrew (Blarina brevicauda)
Source: Front Cell Infect Microbiol. 2020 Sep 9;10:460. doi: 10.3389/fcimb.2020.00460 (PMC7509084; doi:10.3389/fcimb.2020.00460)

Supplementary Material

## Supplementary Figures

**Supplementary Table 1.** Oligonucleotide primers used for amplification and sequencing of RPLV.

| **Segment** | **Primer Name: Oligonucleotide Sequence** |
| --- | --- |
| **S** | OSM55F: 5'-TAGTAGTAGACTCC-3' |
|  | HTN-S1F: 5'-TAGTAGTAGACTYCNTRMDDA-3' |
|  | RPL-S504F: 5'-GNATTMGAYTNAARGATGA-3' |
|  | Han-S604F: 5'-GCHGADGARHTNACACCNGG-3' |
|  | Han-S694F: 5'-CCNGTNATGRGNGTNATHGGNTT-3' |
|  | Cro2F: 5'-AGYCCNGTNATGRGWGTNRTYGG-3' |
|  | RPL-S700F: 5'-ATGGCWKCAAARTGGGARGA-3' |
|  | Han-S952F: 5'-TGGDTNTTTGCNDRDGSNCC-3' |
|  | RPL-S987F: 5'-AAGTNGCWGAAGATGCWGG-3' |
|  | RPL-S1042F: 5'-CAGGAYATGAGNAAYAANAT-3' |
|  | RPL-S1199F: 5'-TGGGGNAAAGAGGCAGTGGA-3' |
|  | RPL-S473R: 5'-ACCCCTAGTTGTTATCATGTA-3' |
|  | HTN-S6R: 5'-AGCTCNGGATCCATNTCATC-3' |
|  | Cro2R: 5'-ANGAYTGRTARAANGANGAYTTYTT-3' |
|  | RPL-S522R: 5'-TCATCYTTNARTCKAATNC-3' |
|  | Hanta-S587R: 5'-CCWGGTGTNANYTCDTC-3' |
|  | SO-S620R: 5'-CCAGGWGTAATCTCWTCAGCC-3' |
|  | RPL-S754R: 5'-CWGTNGCTTTNTCYTCCCATT-3' |
|  | SO-S970R: 5'-GGYSCACHNGCAAANACCCA-3' |
|  | Han-S974R: 5'-TCNGGNGCHCHNGCAAANAHCCA-3' |
|  | Han-S1260R: 5'-CDGGRTCCATRTCATCHCCNA-3' |
|  | RPL-S1062R: 5'-ATNTTRTTNCTCATRTCCTG-3' |
|  | Han-S3R: 5'-TAGTAGTANNCTCCYTRWACA-3' |
| **M** | OSM55F: 5'-TAGTAGTAGACTCC-3' |
|  | M-900F: 5'-CCTMRGGGAGAAGAYCAYGA-3' |
|  | HTN-M1190F: 5'-GGNCCNGGDGCWNVHTGTGA-3' |
|  | RPLV-M1434F: 5'-GGTTTYCATGGKTGGGCAACA-3' |
|  | HTN-M2570F: 5'-GAYACHYTNNTNTTYYTNGG-3' |
|  | HTN-M2620F: 5'-CAATGGTGYWCHASNAMNTG-3' |
|  | RPLV-M-2862F: 5'-TGAATGGTSYGAYCCWGATGG-3' |
|  | RPM-3077F: 5'-GCATGTGATTCWGCAATRTGTTA-3' |
|  | Han-M1482R: 5'-CCATGNANNCCNGGNACACA-3' |
|  | RPLV-M-2339R: 5'-CRGGRCAATCWSCAGGATTAC-3' |
|  | HTM-2409R: 5'-CCACANGCWGTRCANCCWGT-3' |
|  | Han-M2631R: 5'-CATNAYRTCNCCRGGRTCNCC-3' |
|  | Han-M2957R: 5'-GARCCCCANGCNCCNTCWAT-3' |
|  | 7TM-3endR: 5'-TAGTAGTAKRCWCCGCARGAA-3' |
| **L** | OSM55F: 5'-TAGTAGTAGACTCC-3' |
|  | PHL-173F: 5'-GATWAAGCATGAYTGGTCTGA-3' |
|  | RPLV-L-366F: 5'-RGTCACTGTGACAGYWGATGT-3' |
|  | SO-L496F: 5'-TATNGAGTTTGATGTYATTGCTGT-3' |
|  | RPLV-L-655F: 5'-TKCATTGGARGCRATGTTCAAT-3' |
|  | SO-L1060F: 5'-ATGAAATTAGGYAATGCTGA-3' |
|  | 363L-1600F: 5'-TATCCGAGAGCTTGATAGCAC-3' |
|  | Han-L1900F: 5'-ATGAARNTNTGTGCNATNTTTGA-3' |
|  | PHL-2111F: 5'-CAGTCWACARTTGGTGCAAGTGG-3' |
|  | Han-L2520F: 5'-ATNWGHYTDAARGGNATGTCNGG-3' |
|  | HAN-L-F1: 5'-ATGTAYGTBAGTGCWGATGC-3' |
|  | HAN-L-F2: 5'-TGCWGATGCHACNAARTGGTC-3' |
|  | Han-L2936F: 5'-ATGTATGTNAGTGCWGATGC-3' |
|  | Han-L3278F: 5'-TTGCNCAYCAYTCNGATGATG-3' |
|  | Han-L3409F: 5'-GARATGTGGAAAASNATGTT-3' |
|  | RPLV-L-3447F: 5'-CARKCAGGMRASYTATACTGGC-3' |
|  | SO-L4133F: 5'-CCTGTNTATGAATATNTAATACC-3' |
|  | SO-L4637F: 5'-GATTCWAGGACATTRAARGAG-3' |
|  | SO-L4680F: 5'-GAYATATCAATACCAGARGTYATGAG-3' |
|  | RPLV-L-5079F: 5'-GATCCTGAARTTCAGTGTGCAG-3' |
|  | RPLV-L-6105F: 5'-AGCTCAGAGGAAACTGAGGT-3' |
|  | RPLV-L-6375F: 5'-CAGAAAGCACATGTTGCAGTA-3' |
|  | SO-L307R: 5'-TGACTTNAATGTYTTNCCTGTTGG-3' |
|  | SO-L340R: 5'-TAGTTGTCTGGKGTCATCTT-3' |
|  | SO-L400R: 5'-ATACCYCTRTCWACATCAGATGT-3' |
|  | RPLV-L-577R: 5'-MCCGKCATTHCGYCTACTWGGC-3' |
|  | 363L-1050R: 5'-AACTCACTCAACATGTCTCTG-3' |
|  | SL-1458R: 5'-AKTANATGSCCTATATGCCATGC-3' |
|  | SO-L2540R: 5'-CCTGACATACCCTTYARTGA-3' |
|  | SO-L2290R: 5'-TTNARNGCCCATTCNACAGTCTC-3' |
|  | HAN-L-R1: 5'-AACCADTCWGTYCCRTCATC-3' |
|  | HAN-L-R2: 5'-GCRTCRTCWGARTGRTGDGCAA-3' |
|  | PHL-2818R: 5'-GGWCCATAWGAAATGTACTCTTC-3' |
|  | Han-L2970R: 5'-CCNGGNGACCAYTTNGTDGCATC-3' |
|  | SO-L3063R: 5'-AATGCRTCAATCACACANTTYTT-3' |
|  | RPLV-L-4837R: 5'-CYGTMCCYTCWACATTACCTTG-3' |
|  | Han-L5458R: 5'-ATTNGGTTTNCYCCAYTC-3' |
|  | Han-L5621R: 5'-TRTGRWANGCATGTGCAN-3' |
|  | Han-L5754R: 5'-GCWGGNATNCYYTTCATRAA-3' |
|  | Han-L5830R: 5'-TTRAACATRAANARRTCNAC-3' |
|  | Han-L6169R: 5'-GWNACCCNRCTYTTNAT-3' |
|  | Han-L6225R: 5'-TCNCCCCANCCYYTNACWA-3' |
|  | PHL-3endR: 5'-TAGTAGTAGTATGCACCGGAA-3' |
|  | Han-L3R: 5'-TAGTAGTAKGCTCCGNRR-3' |

Abbreviations: A, Adenine; B, C or G or T; C, Cytosine; D, A or G or T; G, Guanine; H, A or C or T; I, Inosine; K, G or T; M, A or C; N, any nucleotide; R, A or G; S, G or C; T, Thymine; V, A or C or G; W, A or T; Y, C or T.

**Supplementary Table 2.** GenBank accession numbers for *Hantaviridae* sequences used in this study.

| **Virus** | **S** | **M** | **L** |
| --- | --- | --- | --- |
| AAIV | DQ345764 | AY515599 |  |
| ALTV |  |  | EU424341 |
| AMRV | AB620028 | AB620029 | AB620030 |
| ANAV | DQ451829 |  |  |
| ANDV | NC003466 | NC003467 | NC003468 |
| ANJV |  | KC490920 |  |
| ARAUV | HQ337907 | FJ409557 |  |
| ARTV | KF974360 | KF974359, MG913807 | EU424339 |
| ARRV | EF650086 |  | EF619961 |
| ASAV | EU929070 | NC038274 | EU929076 |
| ASIV | KC880343 | KC880346 | KC880349 |
| AZGV | JF276226 | JF276227 | JF276228 |
| BCCV | L39949 | L39950 | L39951 |
| BLLV | U19303, U19301 | U19305 |  |
| BLRV |  | AF030551 |  |
| BMJV | AF482713 | AF028025 |  |
| BOGV |  | JX990966 |  |
| BOWV | KC631782 | KC631783 | KC631784 |
| BRGV |  | MF683845 | MF683846 |
| CADV | DQ285566 | DQ284451 |  |
| CATV | DQ256126 | DQ177347 |  |
| CBNV | EF543524 | NC034474 | EF543525 |
| CHOV | DQ285046 | DQ285047 | EF397003 |
| DBSV | AB027523 | AB027115 | DQ989237 |
| DOBV | GQ205407 | GQ205409 | GU904039 |
| ELMCV | AB620106 | AB620104 | AB620108 |
| HOKV | AB010730 |  |  |
| HOLGV | MG599942 | MG599941 | MG599940 |
| HTNV | NC005218 | NC005219 | NC005222 |
| HUPV | JX473273 |  | JX465369 |
| ITPV | DQ345765 | AY515600 |  |
| ISLAV | U19302 | U19304 |  |
| JABV | JN232078 |  |  |
| JJUV | HQ663933 | HQ663934 | HQ663935 |
| JMSV | FJ686859 | FJ593500 | FJ593501 |
| KHAV | U35255 | AJ011648 | AJ011650 |
| KKMV | GQ306148 | KJ857337 | GQ306150 |
| KMJV | JX193698 | JX193699 |  |
| LAIV |  | KM102248 |  |
| LANV | AF005727 | AF005728 | AF005729 |
| LECV | AF482714 | AF028022 |  |
| LHEV | JX465404 | JX465390 | JX465370 |
| LQUV | JX465413 | JX465396 | JX465379 |
| LSCV | AF307322 | AF307323 |  |
| LXV | HM756286 | HM756287 | HQ404253 |
| MJNV | EF641805 | EF641799 | EF641807 |
| MOYV |  |  | JQ287716 |
| MPRLV | AY267347 | AY363179 | EU788002 |
| MTNV | AB620100 | AB620101 | AB620102 |
| MUJV | DQ138142 | DQ138141 |  |
| MULV | U54575 |  |  |
| NVAV | FJ539168 | HQ840957 | FJ593498 |
| NYV | U32591 | U36802 |  |
| ORNV | AF482715 | AF028204 |  |
| OXBV | FJ539166 | FJ539167 | FJ593497 |
| PHV | M34011 | X55129 | EF646763 |
| PRGV | AF482717, EF534079 | AF028028 |  |
| PUUV | EF442087 | NC005223 | NC005225 |
| QDLV | GU566023 | GU566022 | GU566021 |
| QZNV |  | KU950714 |  |
| RIOSV | U18100 |  |  |
| RIOMV | FJ532244 | FJ608550 | FJ809772 |
| RKPV | HM015218 | NC038694 | HM015221 |
| SAAV | AJ616854 | AJ616855 | AJ410618 |
| SANGV | JQ082300 | JQ082301 | JQ082302 |
| SEOV | AF288298 | AB618130 | AF288297 |
| SNV | L33683 | L37903 | L37902 |
| SOOV | AY675349 | AY675353 | DQ056292 |
| SWSV | EF636024 | KY651069 | EF636026 |
| TATV | JX316009 |  |  |
| TGNV |  |  | EF050454 |
| THAIV | AM397666 | AM397671 | JN116258 |
| TIGV |  |  | JQ956487 |
| TOPV | AJ011646 | AJ011647 | AJ011649 |
| TPMV | NC010704 | NC010708 | NC010707 |
| TULV | DQ662090 | DQ665814 | NC005226 |
| ULUV | JX193695 | JX193696 |  |
| VLAV | EU072480 | EU072488 | FJ170807 |
| WEHV | MG599954 | MG599953 | MG599952 |
| WEMBV | MG599945 | MG599944 | MG599943 |
| XSV | KF704709 | KJ000538 | KF704714 |
| XYIV | KF705677 | KF705678 | KF705679 |
| YKSV | JX465423 | NC038705 | JX465389 |
| YUJV | FJ170792 | FJ170800 | FJ170811 |

**Supplementary Table 3.** Sampling localities (state, county) and collection year for *Blarina brevicauda* vouchers housed at the Museum of Southwestern Biology. Tested and positive columns represent the number of specimens analyzed by RT-PCR and showing hantavirus RNA, respectively.

| **State** | **County** | **Year** | **Tested** | **Positive** | **RPLV Strain** |
| --- | --- | --- | --- | --- | --- |
| Arkansas | Washington | 1982 | 1 | 1 | MSB49712 |
| Indiana | Porter | 1994 | 2 | 0 |  |
|  | Westchester | 1994 | 1 | 0 |  |
| Iowa | Allamakee | 1994 | 5 | 1 | MSB73580 |
| Kansas | Douglas | 2001 | 9 | 4 | MSB151832, MSB151836, MSB151838, MSB151840 |
|  | Riley | 1998 | 1 | 0 |  |
| Maryland | Charles | 1997 | 3 | 1 | MSB92373 |
| Massachusetts | Franklin | 1982 | 12 | 7 | MSB47876, MSB47879, MSB47882, MSB47883, MSB47887, MSB47890, MSB47891 |
| Michigan | Leelanau | 1991 | 1 | 0 |  |
|  | Benzie | 1994 | 1 | 0 |  |
|  | Crawford | 1999 | 1 | 1 | MSB92437 |
| Minnesota | Cass | 1983 | 3 | 1 | MSB53254 |
|  | Clay | 1983 | 2 | 2 | MSB53249, MSB53251 |
|  | Morrison | 1998 | 12 | 9 | MSB89858, MSB89859, MSB89861, MSB89862, MSB89863, MSB89864, MSB89866, MSB898867, MSB90845 |
| New Hampshire | Grafton | 1998 | 4 | 1 | MSB151869 |
| New York | Essex | 2001 | 5 | 0 |  |
| Ohio | Ashtabula | 1980 | 1 | 1 | MSB43407 |
|  | Summit | 2001 | 6 | 1 | MSB151834 |
| Pennsylvania | Westmoreland | 1983 | 5 | 1 | MSB53264 |
|  | Indiana | 2001 | 9 | 1 | MSB151849 |
| Tennessee | Monroe | 2001 | 2 | 0 |  |
| Virginia | Fauquer | 1994 | 1 | 1 | MSB76255 |
|  | Page | 1994 | 1 | 0 |  |
|  | Appomattox | 1995 | 1 | 0 |  |
| West Virginia | Wayne | 2001 | 3 | 0 |  |
| Wisconsin | Dane | 2000 | 7 | 3 | MSB151873, MSB151874, MSB151878 |
| Florida | Leon | 1983 | 10 | 1 | MSB53277 (*Blarina carolinensis*) |

**Supplementary Table 4**. GiRaF results with taxa (ID numbers corresponding to voucher specimens at the Museum of Southwestern Biology [MSB]) identified as the result of reassortment between the M and L segment of *Hantaviridae* and the associated percent confidence level of each identified set. Other hantaviruses identified as being possible reassortants are referenced by their abbreviation.

| **Candidate ID** | **Taxa** | **Confidence** |
| --- | --- | --- |
| 17 | MSB89866 | >0.99 |
| 21 | MSB53254, MSB89858, MSB89859, MSB89861, MSB89867 | >0.99 |
| 25 | MSB89861 | >0.99 |
| 30 | MSB89861, MSB89866 | >0.99 |
| 41 | MSB53254, MSB89858, MSB89859, MSB89867 | >0.99 |
| 89 | AMRV, ANDV, ASAV, ASIV, AZGV, BOWV, BRGV, CBNV, CHOV, DBSV, DOBV, ELMCV, HTNV, JJUV, JMSV, KKMV, LANV, LHEV, LQUV, LXV, MJNV, MSB151834, MSB47876, MSB47879, MSB47883, MSB49712, MSB53274, MTNV, NVAV, OXBV, PHV, PUUV, QHSV, RIOMV, RKPV, SANGV, SEOV, SNV, SOOV, SWSV, TPMV, TULV, VLAV, WEHV, WEMBV, XSV, XYIV, YKSV, YUJV | >0.99 |
| 105 | MSB53251 | >0.99 |
| 121 | MSB151838, MSB53251 | >0.99 |
| 127 | MSB151834, MSB151836, MSB151874, MSB151878, MSB47876, MSB47879, MSB47883, MSB53254, MSB53274, MSB73580, MSB89858, MSB89859, MSB89861, MSB89863, MSB89864, MSB89866, MSB89867 | >0.99 |
| 258 | MSB151834, MSB151836, MSB151874, MSB151878, MSB47876, MSB47879, MSB47883, MSB53254, MSB53274, MSB73580, MSB89858, MSB89859, MSB89861, MSB89864, MSB89867 | >0.99 |
| 260 | MSB151834, MSB151836, MSB151874, MSB151878, MSB47876, MSB47879, MSB47883, MSB53254, MSB53274, MSB73580, MSB89858, MSB89859, MSB89861, MSB89864, MSB89867, OXBV | >0.96 |

Abbreviations: AMRV, Amur virus; ANDV, Andes virus; ASAV, Asama virus; ASIV, Asikkala virus; AZGV, Azagny virus; BOWV, Bowé virus; BRGV, Bruges virus; CBNV, Cao Bằng virus; CHOV, Choclo virus; DBSV, Dabieshan virus; DOBV, Dobrava-Belgrade virus; ELMCV, El Moro Canyon virus; HTNV, Hantaan virus; JJUV, Jeju virus; JMSV, Jemez Springs virus; KKMV, Kenkeme virus; LANV, Laguna Negra virus; LHEV, Lianghe virus; LQUV, Lóngquán virus; LXV, Lúxī virus; MJNV, Imjin virus; MTNV, Montano virus; NVAV, Nova virus; OXBV, Oxbow virus; PHV, Prospect Hill virus; PUUV, Puumala virus; QHSV, Qian Hu Shan virus; RIOMV, Rio Mamore virus; RKPV, Rockport virus; SANGV, Sangassou virus; SEOV, Seoul virus; SNV, Sin Nombre virus; SOOV, Soochong virus; SWSV, Seewis virus; TPMV, Thottapalayam virus; TULV, Tula virus; VLAV, Vladivostok ~~Fusong~~ virus; WEHV, Wenling hagfish virus; WEMBV, Wenling minipizza batfish virus; XSV, Xuân Sơn virus; XYIV, Xinyi virus; YKSV, Yákèshí virus; YUJV, Yuanjiang virus.

**Supplementary Table 5**. RDP4 results. Sequences identified as the result of reassortment and the

associated *p*-value of each test. NS indicates that the method did find the breakpoint significant.

| **Recombinant Sequence** | **RDP** | **GENECONV** | **Bootscan** | **Maxchi** | **Chimaera** | **SiSscan** | **3Seq** |
| --- | --- | --- | --- | --- | --- | --- | --- |
| MSB89863_Minnesota | 1.07E-33 | 2.62E-25 | 4.52E-21 | 2.55E-20 | 0.00862061 | 4.11E-42 | 7.23E-39 |
| MSB89861_Minnesota | 4.34E-26 | 5.29E-20 | 2.45E-23 | 5.47E-12 | 1.33E-12 | 6.53E-21 | 1.80E-38 |
| MSB89866_Minnesota | 4.84E-21 | 3.96E-18 | 3.65E-21 | 8.99E-10 | 1.79E-10 | 7.02E-20 | 4.64E-19 |
| MSB47887_Massachusetts | NS | NS | NS | 1.41E-12 | 8.82E-07 | 3.17E-06 | 6.96E-07 |
| MSB49712_Arkansas | NS | NS | NS | 1.20E-05 | NS | 1.47E-07 | 0.03546156 |

**Supplementary Table 6**. RDP4 identified breakpoints for each reassortant sequence.

| **In Alignment** |  | **In Recombinant Sequence** |  | **Recombinant Sequence** |
| --- | --- | --- | --- | --- |
| Begin | End | Begin | End |  |
| 346 | 1035 | 346 | 1025 | MSB89863_Minnesota |
| 1244 | 338 | 1024 | 338 | MSB89861_Minnesota |
| 1422 | 343 | 1300 | 343 | MSB89866_Minnesota |
| 1035 | 1396 | 679 | 1016 | MSB47887_Massachusetts |
| 106 | 348 | 106 | 348 | MSB49712_Arkansas |

**Supplementary Figure 1**. Tanglegram for the M and L segments across the diversity of *Hantaviridae* showing reassortment between the two segments. Sequences representing RPLV are highlighted.


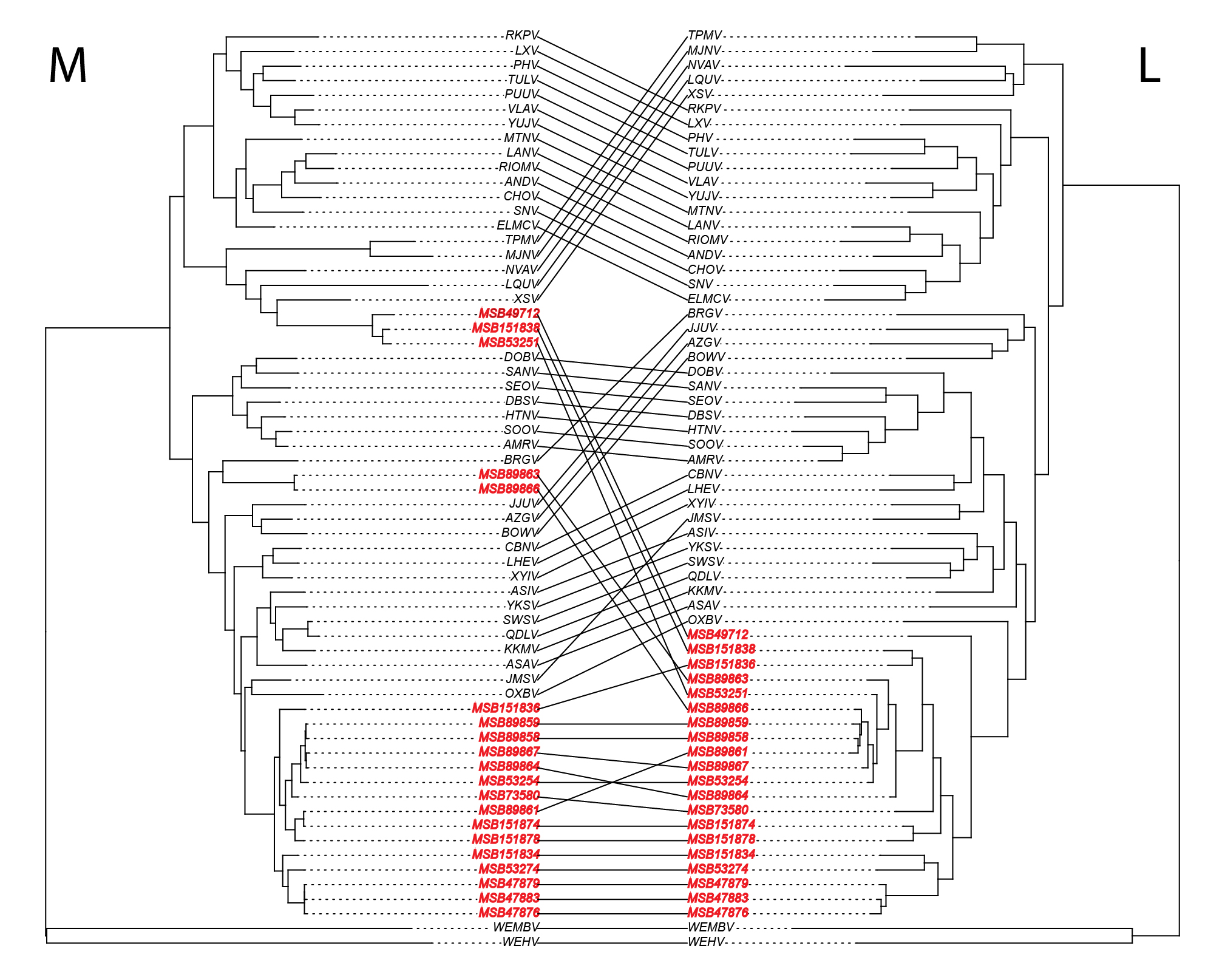

Supplement: Supplementary file 1 [file Data_Sheet_1.docx]
